# Supplementary figures and images for: Increased mTOR Signaling and Impaired Autophagic Flux Are Hallmarks of SARS-CoV-2 Infection
Source: Curr Issues Mol Biol. 2022 Dec 31;45(1):327–36. doi: 10.3390/cimb45010023 (PMC9858158; doi:10.3390/cimb45010023)

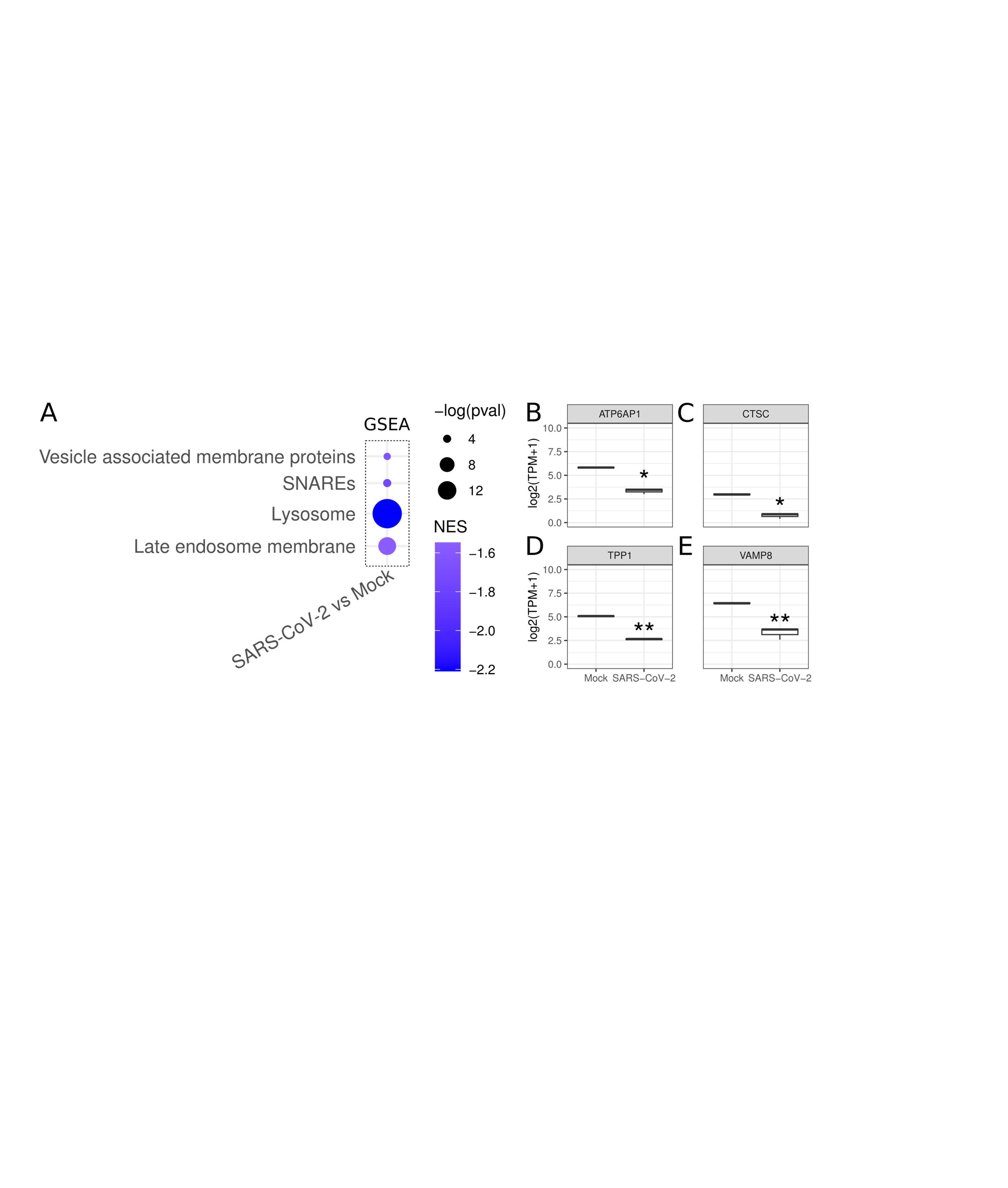

Supplement: Supplementary file 1 [file cimb-45-00023-s001.zip › supplementary materials/figure s1.tif]

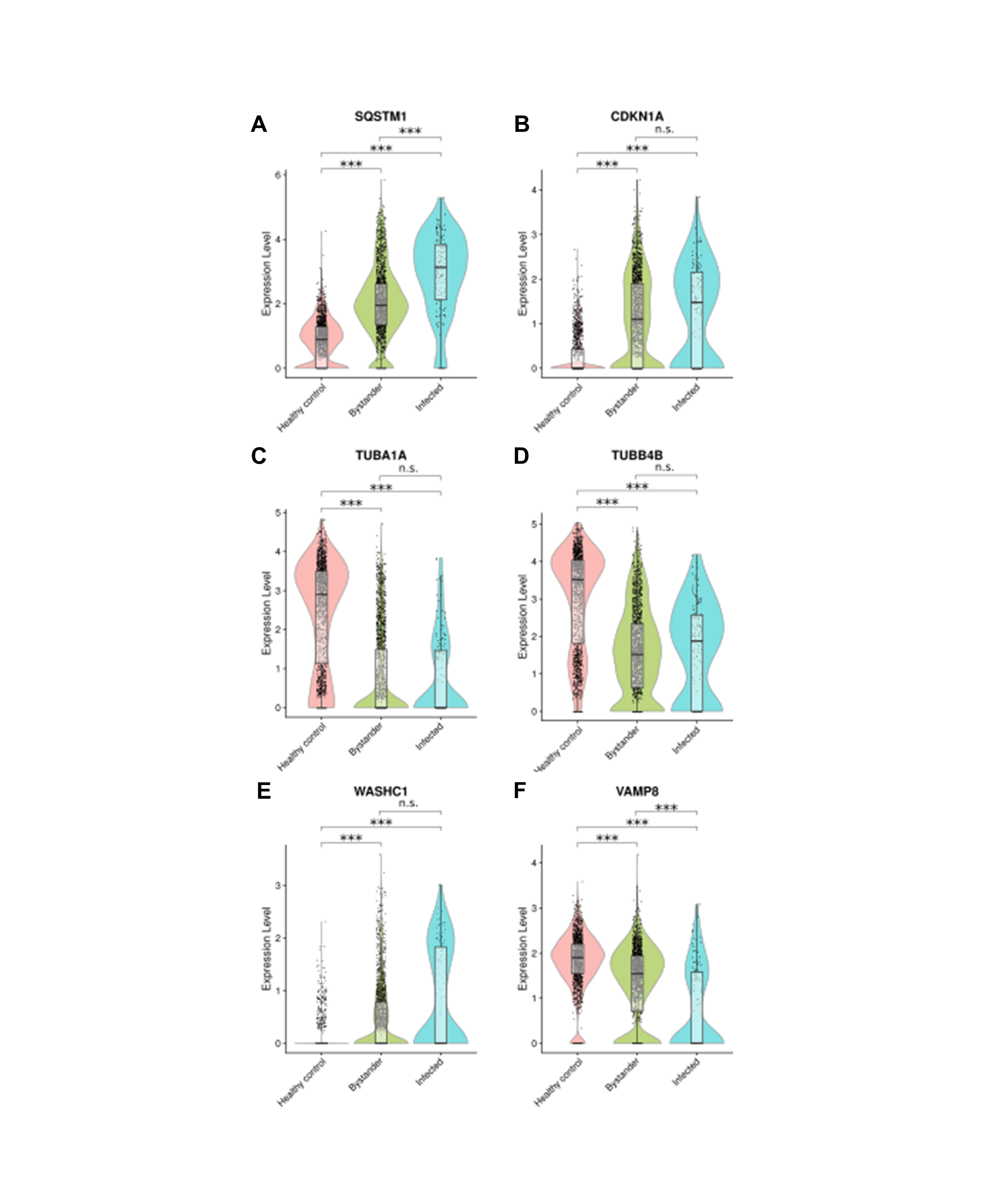

Supplement: Supplementary file 1 [file cimb-45-00023-s001.zip › supplementary materials/Figure s2.tif]
